# A meta-analysis of experimentally validated neo-epitopes: patterns, biases, and opportunities

**DOI:** 10.1007/s00262-025-04209-7

**Published:** 2025-11-06

**Authors:** Alessandro Sette, Ibel Carri, Daniel Marrama, Angela Frentzen, Jarjapu Mahita, Nina Blazeska, Randi Vita, Morten Nielsen, Yat-Tsai Richie Wan, Hannah Carter, Stephen Schoenberger, Bjoern Peters, Zeynep Koşaloğlu‐Yalçın

**Affiliations:** 1 Center for Vaccine Innovation, La Jolla Institute for Immunology, La Jolla, San Diego, CA USA; 2 Department of Medicine, University of California San Diego, La Jolla, San Diego, CA USA; 3 Department of Health Technology, Technical University of Denmark, Lyngby, Denmark; 4 Laboratory of Cellular Immunology, La Jolla Institute for Immunology, La Jolla, San Diego, CA USA

**Keywords:** Neoepitopes, Neoantigens, Immunotherapy, Meta-analysis

## Abstract

Cancer cells harbor somatic mutations that generate novel amino acid sequences that are absent in the self-proteome. These mutation-derived cancer-specific peptides are defined as “neo-peptides”. Neo-peptides eliciting immune responses, i.e. immunogenic neo-peptides, are defined as “neo-epitopes”. Given their relevance to cancer immunotherapy, we conducted a meta-analysis to examine how experimental evidence informs our understanding of neo-epitopes. Our study is the largest reported to date. Using the cancer epitope database and analysis resource (CEDAR), we analyzed over 16,000 neo-peptides tested in more than 20,000 T cell assays across 180 studies. We found that validated neo-epitope frequencies varied across cancer types, with the highest rates in skin and lung and the lowest in colorectal cancer. Neo-epitopes were enriched in driver genes such as TP53 and KRAS. However, testing frequency correlated with mutation prevalence, revealing bias toward recurrent mutations. Despite the high sequence similarity among RAS family members, validated neo-epitope overlap was minimal, challenging pan-RAS strategies. Shared neo-epitopes across cancer types are rare, with only 16 validated in more than one cancer type. While most assays involved HLA class I, class II alleles presented a higher proportion of validated neo-epitopes. Specific alleles, including HLA-B*40:01 and HLA-DRB1*11:01, were enriched for neo-epitopes, whereas others, like HLA-A*02:01, were enriched for non-immunogenic neo-peptides. Finally, amino acid substitutions that altered hydrophobicity or charge were more common in neo-epitopes. Together, these findings define key features of neo-epitopes, expose methodological and biological biases in the literature, and highlight opportunities to improve the selection and prioritization of neo-epitopes for cancer immunotherapy.

**Supplementary Information:**

The online version contains supplementary material available at 10.1007/s00262-025-04209-7.

## Introduction

Cancer cells harbor somatic mutations that create novel amino acid sequences distinct from self-proteins and absent from the germline-derived proteome (self-proteome). We define these mutation-derived cancer-specific peptides as “neo-peptides”. Some neo-peptides are processed, presented on major histocompatibility complex (MHC) molecules, and recognized by T cells, initiating an antitumor immune response. We define these neo-peptides that trigger an immune response, i.e., are immunogenic, as “neo-epitopes”.

In most studies, neo-peptides are identified by analyzing tumor and matched normal sequencing data to detect somatic mutations, which are then translated into altered protein sequences. Candidate neo-peptides are typically filtered and ranked using tools like MHC binding predictions. Selected peptides are synthesized and evaluated in experimental assays to assess their capacity to activate T cells. The distinction between neo-peptides and neo-epitopes is often blurred; we refer to neo-peptides as any peptides derived from somatic mutations, irrespective of immunogenicity. We classify neo-peptides tested and not recognized by T cells as negatives while those eliciting a T cell response as validated neo-epitopes or positives. Note that the same neo-peptide may be tested in multiple contexts, such as T cells from different patients or readouts from different assays, and a single positive response classifies it as a neo-epitope.

Somatic mutations in cancer can occur randomly, but specific genes, known as driver genes, are recurrently mutated because they contribute to tumor initiation and progression [1]. Neo-epitopes derived from driver gene mutations are particularly important because these mutations are typically clonal, highly expressed, and preserved across tumor cells and cancer patients.

Neo-peptides can result from various mutation types, with single nucleotide variants (SNVs) being the most common [2]. SNVs do not disrupt the open reading frame (ORF) and typically produce well-defined neo-peptides. In contrast, neo-peptides derived from frameshift mutations, gene fusions, and RNA alterations such as alternative splicing, intron retention, RNA editing, and the translation of non-coding regions are more complex and challenging to annotate consistently. Based on these considerations, our analysis focused on neo-peptides derived from SNVs.

Unlike self-peptides such as tumor-associated antigens, neo-epitopes are highly tumor-specific and may bypass central tolerance, making them particularly attractive targets for personalized immunotherapies [3]. Despite their therapeutic potential, the landscape of neo-epitopes remains incompletely characterized, with existing studies often focused on individual examples and employing heterogeneous methodologies. While these studies have provided important insights, a systematic synthesis across datasets is needed to uncover broader patterns.

To address this need, we conducted a meta-analysis of experimentally tested neo-peptides using the Cancer Epitope Database and Analysis Resource (CEDAR) [4, 5]. CEDAR is a comprehensive repository that manually curates published data on cancer epitopes and catalogs experimental outcomes from T cell, B cell, and MHC assays. As of January 2025, CEDAR contains 16,602 neo-peptides. This extensive dataset, while reflecting the field’s progress, underscores the necessity for meta-analyses to extract meaningful insights from its complexity. Unlike earlier research based on small datasets of neo-epitopes [6] or primarily computational predictions [7], our study is based on a large dataset of experimentally tested neo-peptides and neo-epitopes that have actually been experimentally validated. Our study provides a comprehensive evaluation of neo-epitope features, uncovering prevailing patterns, biases in the literature, and opportunities for improving neo-epitope selection for cancer immunotherapy.

## Results

### Neo-peptide and neo-epitope data in CEDAR

CEDAR is populated by manual curation of the cancer literature by identifying relevant journal articles from PubMed and scrutinizing them for experimental assays testing epitope recognition by an adaptive immune receptor (described in [4, 5]). It captures all assays providing epitope-specific data, including T cell and B cell recognition and MHC Ligand Elution (MHCLE) assays, capturing both positive and negative outcomes, as well as relevant metadata. While the current focus is on human T cell data, future plans include expanding to murine and other animal models.

**Fig. 1:**
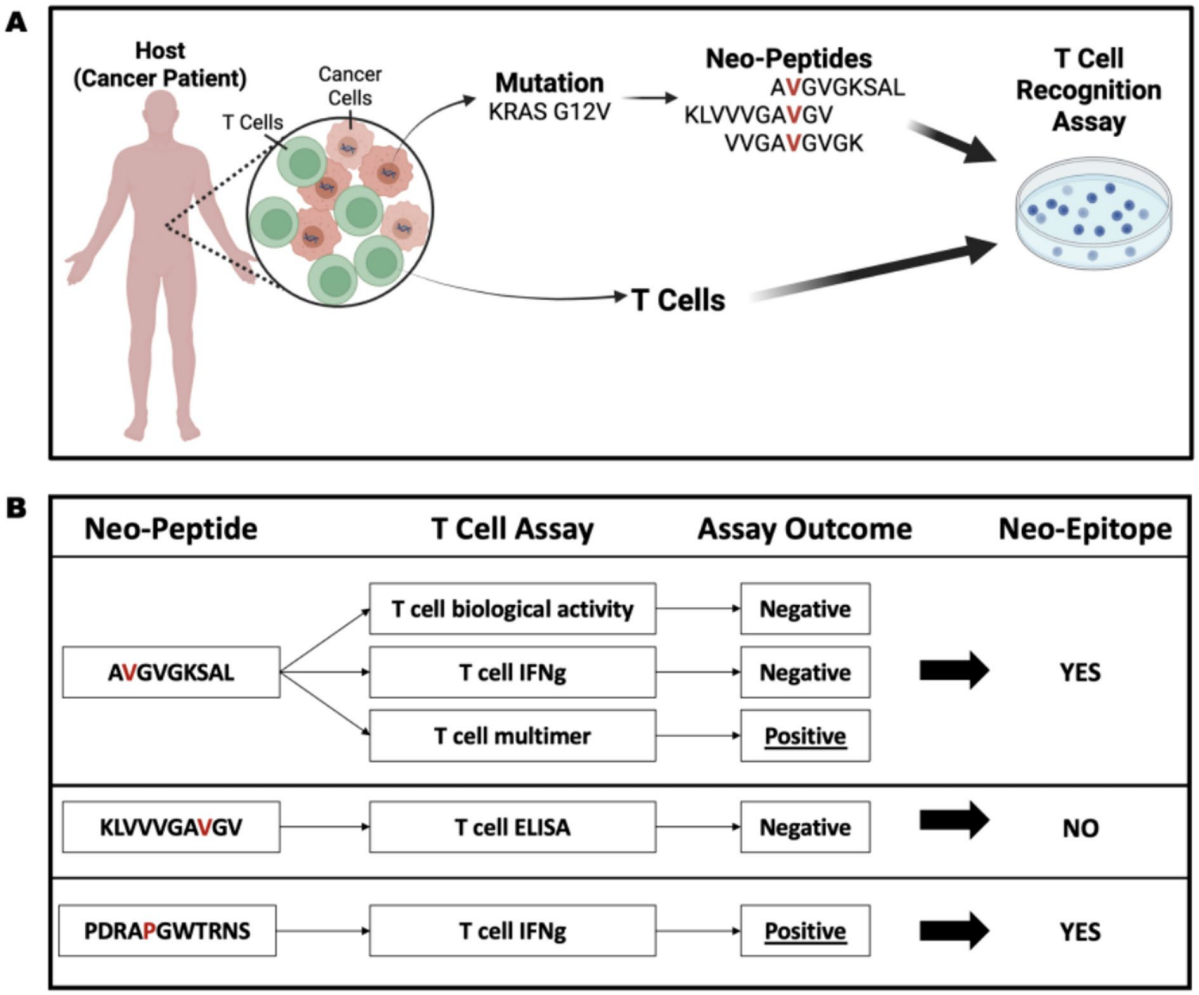
Schematic representation of the data studied in this meta-analysis. **A** This meta-analysis is focused on neo-peptides arising from single nucleotide variations (SNVs) in cancer cells of cancer patients which were tested for recognition by T cells from the same patients. **B** A single peptide may be reported in multiple studies and assessed through various assays, potentially yielding different results. In this study, a neo-peptide was classified as a neo-epitope (“positive”) if at least one T cell assay demonstrated a positive response. Neo-peptides that lacked any positive T cell assay results were categorized as “negative”

This meta-analysis focused on human neo-peptides arising from single nucleotide variations (SNVs) in human cancer patients, which were tested for recognition by T cells from these patients **(**Fig. 1A**)**. To ensure biological relevance and avoid confounding effects, we excluded all assays using T cells from healthy donors or cell lines. This subset includes 16,602 neo-peptides derived from 13,490 unique mutations in 7,887 source proteins tested in 20,601 assays and reported in 180 studies across 28 cancer types (Supplementary Table S1).

A single peptide may be reported in multiple studies and assessed using various assays, potentially yielding different results. A neo-peptide was classified as a neo-epitope (“positive”) if at least one T cell assay showed a positive response **(**Fig. 1B**)**. Neo-peptides that lacked any positive T cell assay results were categorized as “negative”. Following this classification, 2,178 peptides (13%) were classified as neo-epitopes, and 14,424 peptides (87%) were classified as negative neo-peptides (Supplementary Table S2).

We note that data quality and consistency vary considerably across the literature. CEDAR, experimental results are included as reported in the original publications, without applying external quality scoring. While this inclusive approach allows for comprehensive coverage, it also introduces heterogeneity in assay conditions, reporting practices, and metadata availability. To illustrate this variability, we reviewed a small random subset of neo-peptides **(**Supplementary Table S3**)** and found differences in assay type, study overlap, and consistency of outcomes across publications. These examples underscore the challenges in defining a universal ground truth but also highlight the importance of careful interpretation in meta-analyses. To partially address these concerns, we also generated a stricter subset of “repeatedly tested” neo-peptides, defined as peptides with at least three reported assays and classified as neo-epitopes only if they had at least two positive assay results **(**Supplementary Table S2**)**. This reproducibility-based filter reduces the influence of single-study outliers, albeit at the cost of substantially reducing the number of peptides available for analysis. Following this stricter classification, only 518 neo-peptides were retained, of which 301 were classified as neo-epitopes (58%) and 217 were classified as negative neo-peptides (42%). Although this reproducibility filter substantially reduced the dataset, the majority of the retained peptides were classified as positive, suggesting that immunogenic peptides are more likely to be confirmed across studies.

### Assay types utilized to study neo-peptides

We investigated the different experimental methods, i.e., assay types, for evaluating neo-peptides for T cell recognition and found that Enzyme-Linked ImmunoSpot (ELISPOT) assays were the most common, comprising 68% of assays. This was followed by multimer/tetramer binding assays (12%), biological activity assays, such as activation or degranulation assays (10%), enzyme-linked immunosorbent assays (ELISA, 7%), and intracellular cytokine staining (ICS, 2%). Interferon-gamma (IFN-γ) was the most frequently measured cytokine, while other relevant cytokines, including interleukin-5 (IL-5), tumor necrosis factor-alpha (TNF-α), interleukin-2 (IL-2), and interleukin-17 (IL-17), were assessed less frequently (Supplementary Table S4). These findings indicate a potential knowledge gap regarding the scarcity of assays describing the direct recognition and killing of cancer cells and assays determining the multi-specificity of T cell responses.

### Protein size influences the number of neo-peptides but not neo-epitope enrichment

**Fig. 2:**
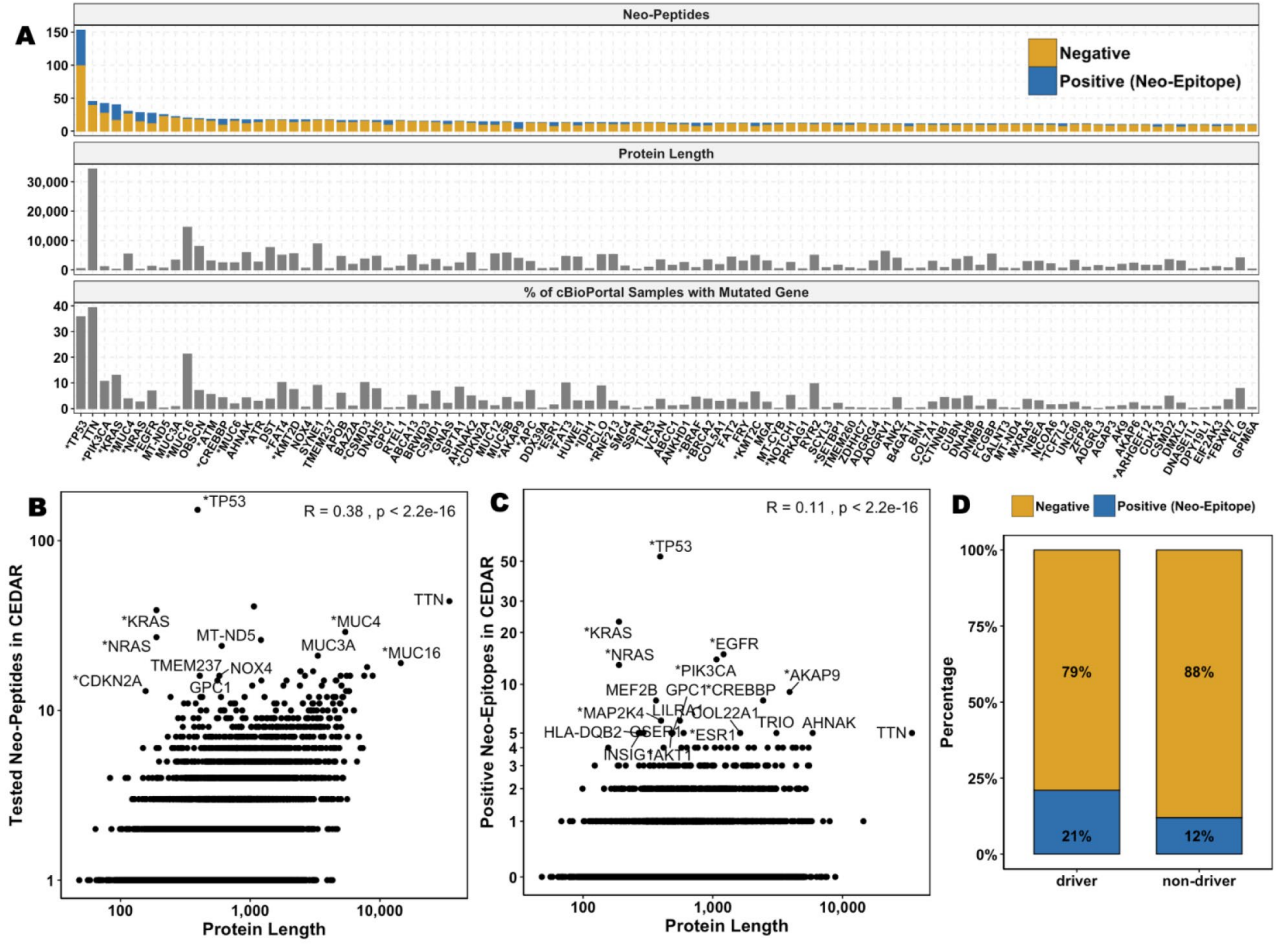
Correlations between Mutational Frequency, Protein Length and Driver Gene Status. **A** Top 100 proteins with the most tested neo-peptides. Driver genes are indicated with *. The top panel shows the number of neo-epitopes (positives, blue) and neo-peptides (negatives, yellow) tested per gene, the middle panel shows the protein length, and the bottom panel shows the percentage of tumor samples from cBioPortal that carry the corresponding mutation. **B** Correlation between the number of tested neo-peptides reported in CEDAR and the source protein length. **C** Correlation between the number of neo-epitopes reported in CEDAR and the source protein length. **D** Percentage of neo-epitopes from the total number of tested neo-peptides found in driver vs. non-driver genes

Among the top 100 proteins with the most tested neo-peptides, many were large or encoded by genes that are highly mutated in tumor samples recorded in cBioPortal (Fig. 2A**, **Supplementary Table S5) [8]. Mutations often result from random errors during DNA replication, making larger genes more likely to harbor mutations simply because they have a greater number of coding nucleotides (extensively reviewed in [9]). We found a strong correlation between the number of tested neo-peptides in CEDAR and protein length (Pearson’s correlation coefficient R = 0.38, *p* < 2.2 × 10⁻^1^⁶, Fig. 2B). We also observed a significant correlation between the number of positive neo-epitopes and protein length; however, the low Pearson’s correlation coefficient (Pearson’s correlation R = 0.12, *p* < 2.2 × 10⁻^1^⁶) indicates that the association is weak (Fig. 2C).

These findings indicate that, although larger proteins accumulate more, mostly random, mutations, leading to more potential neo-peptides, neo-epitopes do not appear to originate primarily from larger proteins. We calculated the fraction of neo-epitopes per tested neo-peptide for each protein and compared values across protein size quartiles. We found that proteins in the smallest quartile (Q1) showed a significantly higher mean neo-epitope fraction (0.1334) compared to those in the largest quartile (Q4, 0.1176, Wilcoxon rank sum test *p* = 5.55 × 10⁻^4^, Supplementary Figure S1). However, the median neo-epitope fraction remained identical (0.01) across both quartiles, suggesting that, while there was an overall trend, the effect size might be small.

### Driver Genes Are Enriched for Neo-Epitopes

Next, we investigated whether neo-epitopes are more likely to originate from proteins encoded by cancer driver genes. Our analysis revealed that driver genes are significantly enriched for neo-epitopes compared to non-driver genes (Fisher’s exact test, *p* < 2.2 × 10⁻^1^⁶; Fig. 2D). To assess if this enrichment is due to protein size, we compared the lengths of proteins encoded by driver and non-driver genes. We found that in our dataset, proteins encoded by driver genes had a median length of 866 amino acids and were significantly longer than proteins encoded by non-driver genes, which had a median length of 585 amino acids (Wilcoxon rank sum test, *p* < 2.2 × 10⁻^1^⁶, Supplementary Figure S2). However, as shown in the previous section, smaller proteins showed a higher mean neo-epitope fraction, suggesting that protein size alone does not explain the observed enrichment. These findings imply that other factors, such as the clonality or expression levels of driver genes, may contribute to their enrichment in neo-epitopes.

### p53 Neo-peptides are most frequently reported and validated as neo-epitopes

TP53 is the most frequently mutated gene in human cancers [10]; hence, it is unsurprising that neo-peptides derived from TP53 mutations are most frequently reported in CEDAR. A total of 152 unique neo-peptides generated by SNVs from 68 unique amino acid mutations at 47 distinct positions of p53 were recorded in CEDAR (Supplementary Table S6**)**. Of the 152 tested neo-peptides, 53 were neo-epitopes (35%). The fraction of neo-epitopes was significantly higher in p53 than the overall fraction of neo-epitopes in the complete dataset (6%, Chi-Squared Test, *p* = 1.51 × 10⁻^14^).

**Fig. 3:**
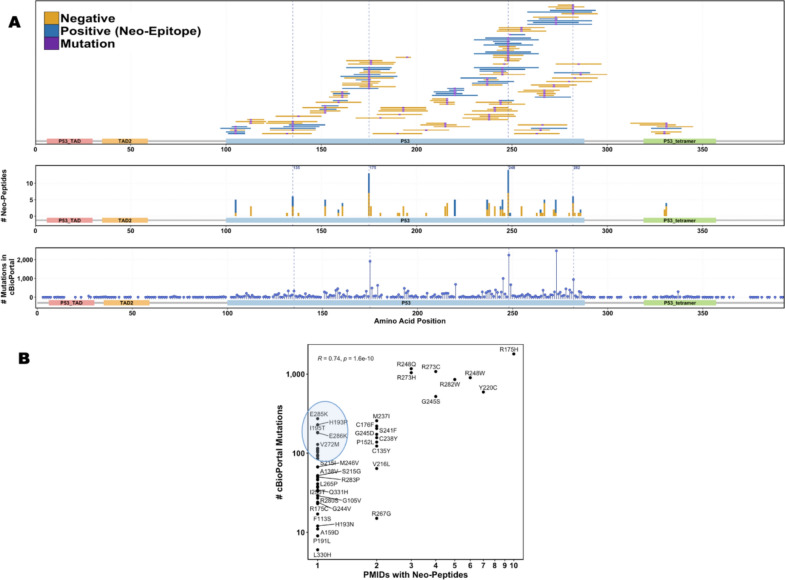
p53 Neo-Peptides and Mutations. **A** The top panel shows the distribution of tested neo-peptides across the p53 protein. The middle panel shows the total number of neo-epitopes (positives) and neo-peptides (negatives) tested per each p53 mutation. The bottom panel shows the number of tumor samples in cBioPortal that contain a somatic mutation in each position of p53. The functional domains of p53 are shown at the bottom of each panel and with abbreviations and positions: P53 transactivation motif (P53_TAD, 6–30), Transactivation domain 2 (TAD2, 35–59), P53 DNA-binding domain (P53, 100–288), P53 tetramerization motif (P53_tetramer, 319–357). Neo-peptides (negatives) are shown in yellow, neo-epitopes (positives) in blue, mutated residue in purple. **B** Correlation between TP53 mutation frequency in tumors from cBioPortal and the number of studies evaluating neo-peptides from these mutations. Mutations observed in over 100 patients, for which neo-peptides were tested in only a single study, are highlighted in blue

The majority of neo-peptides are located in the p53 DNA-binding domain (shown as P53 at positions 100–288 in Fig. 3A), which is central to p53’s function as a transcription factor. Most neo-peptides arise from mutations at positions 135, 175, 248, and 282, which are well-known mutation hotspots [11]. Mutation R175H is the most studied, with 11 unique neo-peptides derived from it, evaluated in 54 different T cell assays across 10 studies, with 34 assays (63%) showing positive responses. Conversely, the majority of p53 neo-peptide mutations (74%) have been poorly studied and reported in only a single study.

We retrieved all p53 missense mutations from cBioPortal and analyzed the relationship between mutation frequency and the number of studies investigating neo-peptides derived from each mutation. Our analysis showed a strong positive correlation between mutation frequency and the number of studies evaluating neo-peptides (Pearson’s correlation coefficient R = 0.74, *p* = 1.6 × 10⁻^1^⁰, Fig. 3B). However, some frequently occurring mutations appear to be understudied; despite being present in over 100 patients in cBioPortal, neo-peptides from those mutations have only been tested in single studies (highlighted in blue in Fig. 3B).

In the stricter subset of repeatedly tested neo-peptides only 17 neo-peptides were retained, of which 16 were neo-epitopes.

### Limited neo-epitope overlap among RAS family proteins

**Fig. 4:**
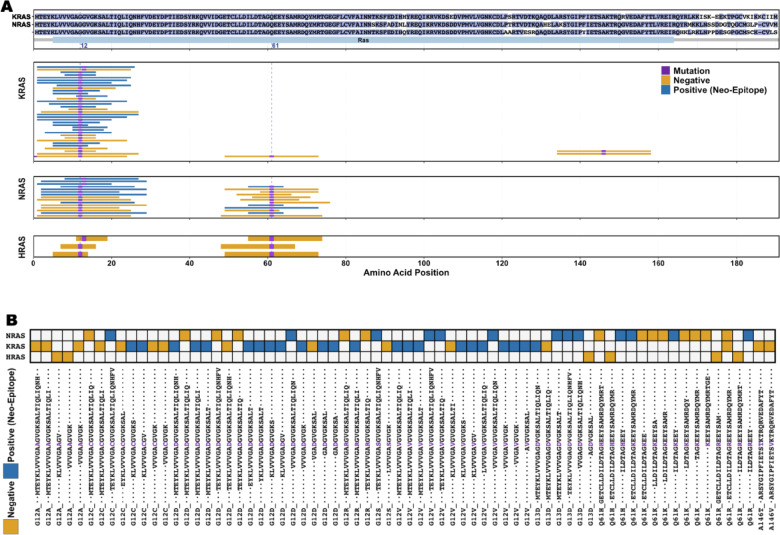
Neo-peptides in the RAS family. **A** The top panel shows the alignment of sequences of the NRAS, HRAS, and KRAS proteins with conserved amino acids highlighted in blue. The lower panels show the distributions of tested neo-peptides across each of the proteins KRAS, NRAS, and HRAS. **B** Shared neo-peptide sequences across KRAS, NRAS, and HRAS. Neo-peptides (negatives) are shown in yellow, neo-epitopes (positives) in blue, mutated residue in purple

The RAS gene family encodes proteins that regulate key cell signaling pathways involved in proliferation, differentiation, and survival. Mutations in KRAS, NRAS, and HRAS are among the most frequent oncogenic alterations in human cancers [12]. While these genes are located at different genomic sites, their encoded proteins have significant sequence similarity, particularly in their functional Ras domain **(**Fig. 4A**,** top panel**)**. This similarity supports a pan-RAS immunotherapy approach aimed at identifying shared neo-epitopes across multiple RAS proteins, enabling targeted therapies for various cancer types and patient populations [13].

We analyzed the neo-peptide data in CEDAR to identify shared neo-peptides and neo-epitopes among KRAS, NRAS, and HRAS. Because the genes are located at different genomic locations, the genomic positions of mutations across the three genes are different, but because the translated protein sequences are highly similar and even identical in many areas, many amino acid mutations occur at equivalent protein positions. A total of 72 Ras neo-peptides were reported in CEDAR, including six from HRAS, 27 from NRAS, and 40 from KRAS (Fig. 4B**, **Supplementary Table S7). Of these, 37 (51%) were validated as neo-epitopes by at least one positive T cell assay. The 72 neo-peptides originated from 22 unique missense mutations, primarily at the hotspot mutation position G12, followed by Q61 (Fig. 4A). Surprisingly, only one shared neo-peptide has been reported: the neo-peptide ETCLLDILDTAG**R**EEYSAMRDQYMR encoded by the mutation Q61R was tested in both, KRAS and NRAS, but did not yield any positive T cell response. Despite being derived from the same amino acid mutations, all other neo-peptides exhibited slight sequence differences (Fig. 4B). These findings indicate that while mutations in the RAS gene family mutations are frequent oncogenic alterations, most neo-peptides exhibit sequence variations, with only one shared neo-peptide identified across KRAS and NRAS, highlighting a potential challenge for a pan-RAS neo-epitope approach.

In the stricter subset of repeatedly tested neo-peptides, 20 peptides were retained, of which 17 originated from mutations at the G12 hotspot. Notably, none of the HRAS-derived neo-peptides met the repeated testing criteria. Among the retained peptides, 16 were derived from KRAS, 13 of which were classified as neo-epitopes, and four were from NRAS, three of which were classified as neo-epitopes.

### Recurrent mutations and author redundancy in neo-epitope studies

The analysis of neo-peptides from p53 and Ras Family proteins revealed an important issue: certain well-known mutations and their associated neo-peptides are over-represented in neo-epitope research. Our dataset includes 16,602 unique neo-peptides that have been tested in 20,601 T cell assays, indicating that while most neo-epitopes were tested only once, some were tested multiple times. Additionally, the 16,602 neo-peptides were derived from 13,490 unique SNVs, indicating that certain mutations led to multiple neo-peptides being tested, possibly across multiple assays.

**Fig. 5:**
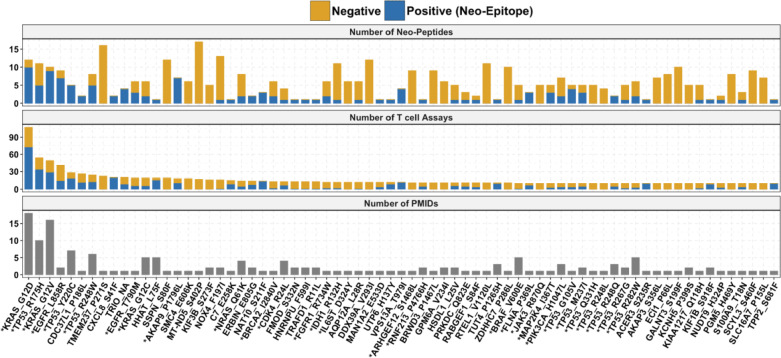
Well-Studied Mutations that were tested in at least 10 assays. Driver genes are indicated with *. The top panel shows the number of neo-epitopes and neo-peptides tested per mutation. The middle panel shows the number of positive and negative T cell assays per mutation, and the bottom panel shows the number of studies that tested the neo-peptides. Neo-peptides (negatives) are shown in yellow, neo-epitopes (positives) in blue

We analyzed the subset of mutations with tested neo-peptides reported in multiple studies and found that only 255 out of the 13,490 mutations (2%) fell into this category (Supplementary Table S8**)**. A significant proportion of these mutations came from driver genes (27%). A select group of mutations was disproportionately studied, with multiple overlapping neo-peptides examined across numerous assays and studies (Fig. 5). Among these, KRAS_G12D, TP53_R175, and KRAS_G12V were the top three in terms of the number of tested neo-peptides, assays performed, and distinct studies, having been analyzed in over ten independent studies.

Of the 255 mutations for which neo-peptides were tested in multiple studies, 213 (83%) shared at least one author, suggesting that these are likely follow-up investigations of initial findings. The remaining 42 mutations were examined in independent studies with non-overlapping authors. Of these independently studied mutations, 27 were from driver genes, and 15 were from non-drivers. This subset is of particular interest because some mutations classified as passenger mutations may recur in multiple patients across different cancer types.

For instance, the mutation VPS16_S404F appeared in two independent studies involving melanoma patients, testing five overlapping neo-peptides, with two showing positive T cell assay outcomes. These findings highlight the CEDAR’s value in uncovering neo-peptide recurrence patterns across studies. By capturing data granularity, CEDAR enables differentiation between independent discoveries and follow-up investigations through metadata such as author lists.

To extend this analysis, we analyzed author overlap across studies that tested the same mutations. Among all combinations of studies evaluating the same mutations, we identified 63 unique study groups, defined by distinct combinations of PubMed IDs (Supplementary Figure S3**, **Supplementary Table S9). For each group, we quantified the number of shared authors and the number of distinct mutations tested. The number of shared authors across study groups ranged from 1 to 21, with a median of 5, indicating that many investigations were driven by recurring sets of collaborators.

### Neo-epitope landscape across cancer types

**Fig. 6:**
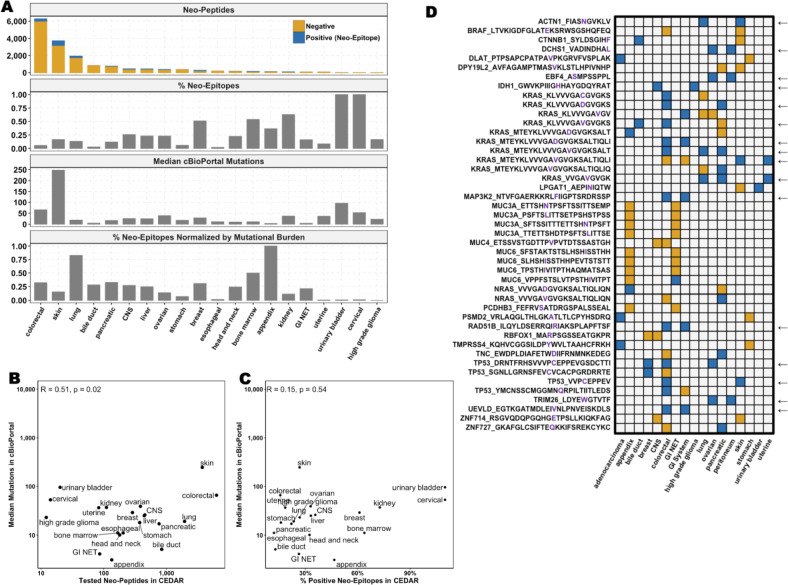
Neo-Peptides and Neo-Epitopes per Cancer Type. **A** The top panel shows the number of neo-epitopes (positives) and neo-peptides (negatives), the second panel shows the percentage of neo-epitopes among all tested neo-peptides, the third panel shows the median of mutations in tumor samples from cBioPortal, and the bottom panel shows the percentage of neo-epitopes normalized by mutation frequency. **B** Correlation between the number of tested neo-peptides in CEDAR and the median number of mutations in cBioPortal per cancer type. **C** Correlation between the percentage of positive neo-epitopes among neo-peptides in CEDAR and the median number of mutations in cBioPortal per cancer type **D** Neo-peptides shared between cancer types. Neo-peptides (negatives) are shown in yellow, neo-epitopes (positives) in blue. An arrow indicates shared neo-epitopes

Next, we investigated the landscape of tested neo-peptides and the proportion that were validated as neo-epitopes across cancer types. We define the “positivity rate” as the fraction of neo-peptides that elicited at least one positive T cell response among all neo-peptides tested for a given cancer type. As expected, common cancers with high mutational burdens, such as colorectal cancer (6,283 neo-peptides), skin cancer (3,725 neo-peptides), and lung cancer (1,959 neo-peptides), were frequently studied **(**Fig. 6A**, **Supplementary Table S10**)**. The positivity rate of colorectal cancer of 6% was significantly lower than that of skin and lung cancer, which demonstrates higher percentages of neo-epitopes of 17 and 13%, respectively (Chi-Squared Test *p* < 2.2 × 10^–16^). These results align with the known responsiveness of skin and lung cancers to immunotherapies [14] and highlight that frequent mutations do not necessarily result in frequent neo-epitope occurrences.

Esophageal and bile duct cancers exhibited the lowest fraction of neo-epitopes, with positivity rates of less than 2 and 3%, respectively. This aligns with their classification as “cold tumors”, characterized by an immunosuppressive microenvironment and limited immune cell infiltration [15, 16]. Despite their overall low immunogenicity, 23 neo-epitopes were identified in bile duct cancer and four in esophageal cancer, indicating that while rare, there are still neo-epitopes recognized by T cells in these tumors. Pancreatic and bile duct cancers are also both classified as “cold tumors” and had a similar number of reported neo-peptides (771 and 840, respectively) [15, 17]. However, pancreatic cancer had a significantly higher proportion of neo-epitopes (12%) than bile duct cancer (3%, Chi-squared Test *p* < 2.2 × 10^–16^).

To explore the relationship between mutational burden and neo-epitope frequency, we analyzed the median number of somatic mutations for each cancer type using cBioPortal [8]. We found a positive correlation between mutational burden and the number of tested neo-peptides (Pearson’s correlation coefficient R = 0.51, *p* = 0.021, Fig. 6B), which is consistent with the expectation that a higher mutational load generates more neo-peptides. However, the correlation between the percentage of neo-epitopes and mutational burden was weak (Pearson’s correlation coefficient R = 0.15, *p* = 0.53, not significant, Fig. 6C), suggesting that more mutations do not necessarily lead to increased T cell recognition.

To account for variations in mutational burden, we normalized the fraction of neo-epitopes by the median number of mutations per cancer type, as detailed in the Methods section (lower panels in Fig. 6A). This adjustment enabled a more accurate comparison of neo-epitope frequencies across tumors with varying mutation rates. After normalization, colorectal cancer’s neo-epitope fraction increased to 39%, indicating that its initially low neo-epitope fraction was due to its high mutation load rather than a fundamental lack of T cell recognition. Lung cancer showed a comparable pattern, further supporting the link between mutation burden and neo-epitope generation. Conversely, skin cancer, despite having the highest mutational burden (247 mutations per tumor), underwent minimal change post-normalization, suggesting that its neo-epitope fraction is not solely driven by mutation frequency but may be influenced by additional factors. Bile duct and pancreatic cancers, which have relatively low mutation rates, showed increased neo-epitope fractions after normalization, implying that despite having fewer mutations, a higher proportion of them may be recognized by T cells.

Lastly, we identified 45 neo-peptides shared between at least two different cancer types; however, only 16 neo-epitopes eliciting immune responses were shared (Fig. 6D**, **Supplementary Table S11). Notably, TP53- and KRAS-derived neo-peptides were among the most frequently shared.

### Patterns of HLA restriction and allelic enrichment in neo-epitope presentation

Restrictions to MHC, or Human Leukocyte Antigen (HLA) in humans, are curated in CEDAR when available. Among the 20,601 assays analyzed, 11,460 reported HLA restriction, with 6,369 providing HLA alleles at four-digit resolution. The majority of assays (83%) focused on HLA class I, underscoring its key role in presenting intracellular peptides to CD8⁺ T cells and its prominence in cancer immunology research [18].

**Fig. 7:**
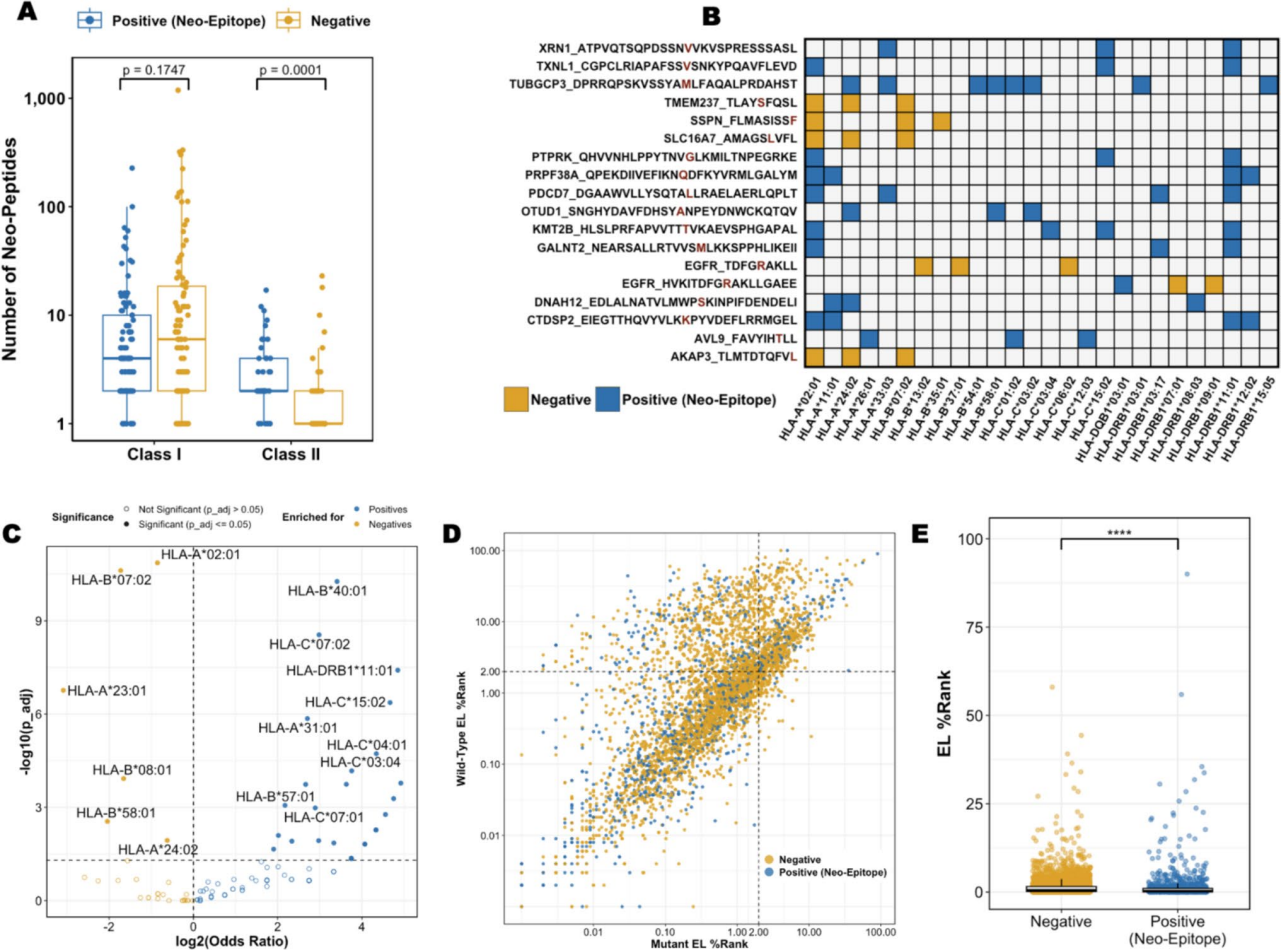
HLA restriction patterns. **A** Number of neo-peptides and neo-epitopes per HLA class. **B** Neo-epitopes and neo-peptides tested in at least 3 different HLAs. **C** Enrichment analysis of neo-epitopes and neo-peptides per HLA. **D** Predicted eluted ligand percentile ranks (EL %-Rank) of mutant and wild-type sequences. **E** Neo-epitopes were predicted to bind significantly stronger than negative neo-peptides. Neo-peptides (negatives) are shown in yellow, neo-epitopes (positives) in blue

The dataset included 143 distinct HLA alleles, including 99 class I and 44 class II allelic variants. Fewer HLA class II alleles were tested, and they had a lower number of peptides per allele compared to class I alleles (Fig. 7A). Specifically, class I alleles had a median of nine peptides tested per allele, ranging from 1 to 1,410 peptides, while class II alleles had a median of two peptides tested per allele, ranging from one to 27. Notably, a significantly higher proportion was positive compared to neo-peptides tested with HLA class I restriction (Wilcoxon test, *p* = 0.0001, Fig. 7A).

We further investigated neo-peptide:HLA pairs to identify overlaps and identified 166 neo-peptides tested in the context of at least two distinct HLA alleles (Supplementary Figure S4; Fig. 7B shows the subset of peptides tested against at least three distinct HLA alleles). Notably, the neo-peptide TUBGCP3_DPRRQPSKVSSYAMLFAQALPRDAHST was presented by eight different HLA molecules and produced positive T cell assay outcomes in every context. In fact, an analysis of peptide length and the number of HLA alleles tested for each peptide showed a positive correlation (Pearson’s correlation coefficient of 0.17, *p*-value = 2.2 × 10⁻^16^, Supplementary Figure S5)**.** This suggests that longer peptides are more likely to be tested across a wider range of HLA alleles, potentially because they can accommodate multiple binding cores, allowing compatibility with various HLA motifs.

Next, we conducted an HLA enrichment analysis to investigate associations between HLA molecules and neo-epitopes using Fisher’s exact test odds ratios (OR) with Benjamini–Hochberg corrected p-values (p-adj; Fig. 7C). We found that 37 of the 143 HLA alleles demonstrated significant enrichment patterns; 31 HLA alleles were significantly enriched for neo-epitopes, while six alleles were enriched for negative neo-peptides. The top three HLA alleles enriched for neo-epitopes were HLA-B*40:01 (OR: 10.63, p-adj = 5.42 × 10⁻^11^), HLA-C*15:02 (OR: 25.42, p-adj = 4.25 × 10⁻⁷), and HLA-DRB1*11:01 (OR: 28.85, p-adj = 3.87 × 10⁻⁸). The top three HLA alleles enriched for negative neo-peptides were HLA-B*07:02 (OR = 0.30, p-adj = 2.41 × 10⁻^11^), HLA-B*08:01* (OR = 0.31, p-adj = 1.19 × 10), and HLA-A*02:01* (OR = 0.55, p-adj = 1.36 × 10⁻^11^).

In summary, our analysis highlights the diversity and bias in HLA representation among neo-peptide studies. Allele-specific enrichment patterns indicated that certain HLA alleles were significantly more likely to be associated with T cell responses to neo-epitopes, suggesting a potential bias or enhanced capacity of these alleles to present immunogenic peptides.

#### HLA binding predictions for neo-peptides and neo-epitopes

Next, we investigated the relationship between HLA-binding and neo-epitope recognition. Our dataset included 5,019 neo-peptide:HLA pairs, of which 4,808 correspond to HLA class I and 190 to class II allelic variants. Focusing on the HLA class I-restricted subset, we predicted binding for each neo-peptide and its corresponding wild-type counterpart using NetMHCpan[19] (**Fig. 2.11 7D, **Supplementary Table S12).

The majority of neo-peptides (81%) were predicted to bind their HLA alleles with eluted ligand percentile ranks below the suggested binding threshold (EL %-Rank < 2); 51% of neo-peptides were classified as strong binders (EL %-Rank < 0.5). With a median EL %-Rank of 0.27, neo-epitopes were predicted to bind significantly stronger than negative neo-peptides, which had a median EL %-Rank of 0.528 (Wilcoxon Test, *p*-value < 2.2 × 10^–16^; Fig. 7E).

Next, we compared EL %-Rank predictions of mutant and wild-type within each group. In both neo-epitopes and negative neo-peptides, mutant sequences exhibited significantly stronger binding than their wild-type counterparts (Wilcoxon test, *p* < 2.2 × 10⁻^1^⁶; Supplementary Figure S6). We calculated the differential agretopicity index (DAI), defined as the difference in binding affinity between mutant and wild-type peptides, to assess its role in neo-epitope recognition [20] and found no significant difference in DAI scores between neo-epitopes and negative peptides (Wilcoxon test, *p* = 0.3056, Supplementary Figure S7).

#### Amino Acid Substitution and Enrichment Patterns

**Fig. 8:**
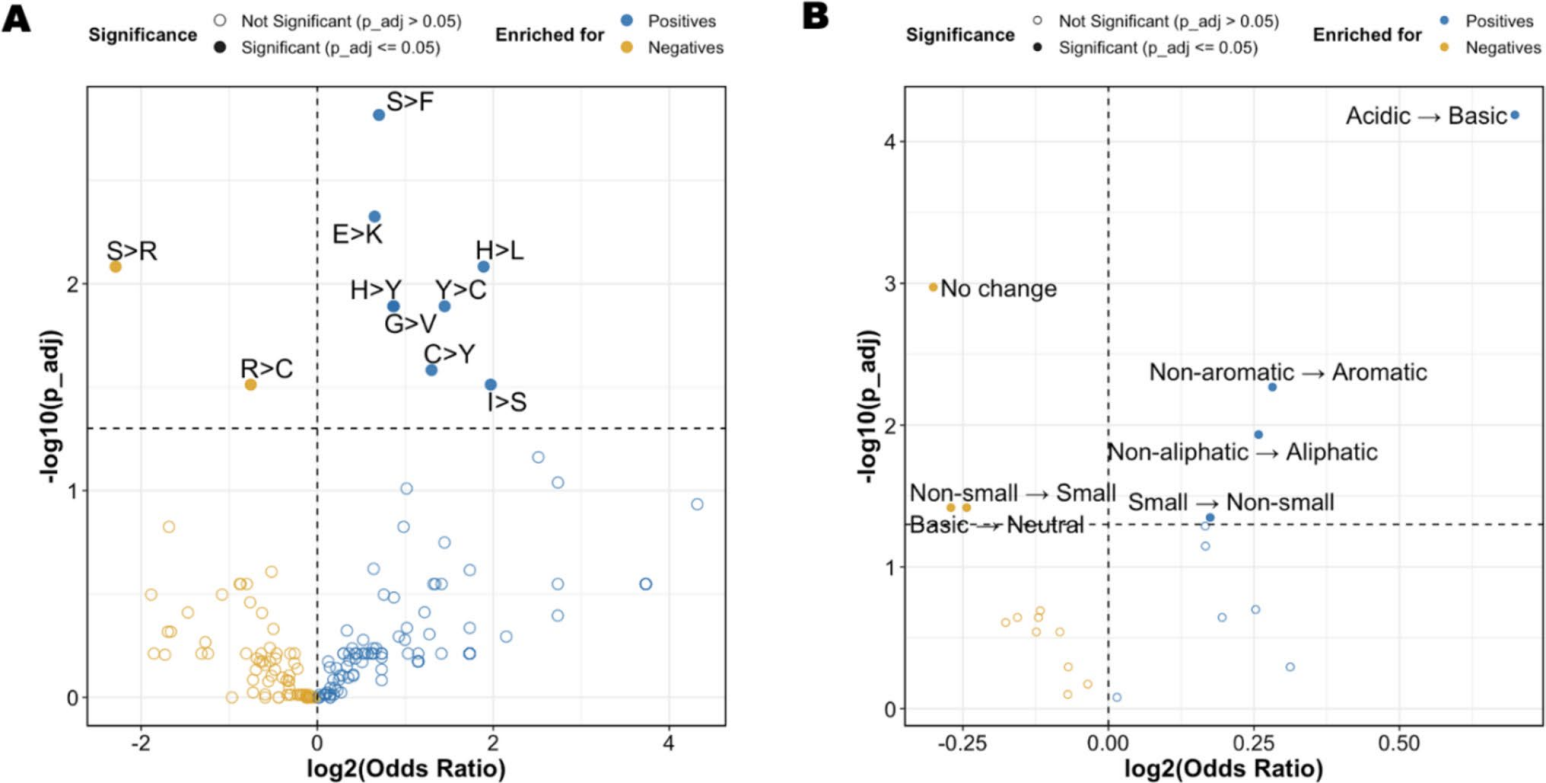
Mutations in neo-peptides and neo-epitopes. **A** Enrichment analysis of amino acid changes. **B** Enrichment analysis of changes in physicochemical properties. Neo-peptides (negatives) are shown in yellow, neo-epitopes (positives) in blue

Biochemical transformations from somatic mutations are crucial for overcoming central tolerance to self-peptides and for enhancing neo-epitope recognition [21, 22]. We conducted an enrichment analysis across all mutation types using Fisher’s exact test odds ratios (ORs) with Benjamini–Hochberg corrected p-values (p-adj) to identify specific mutations linked to increased neo-epitope recognition. Mutations significantly enriched in neo-epitopes included serine to phenylalanine (S → F, OR = 1.63, p-adj = 0.001), glutamic acid to lysine (E → K, OR = 1.57, p-adj = 0.004), and histidine to leucine (H → L, OR = 3.71, p-adj = 0.008; Fig. 8A). Conversely, mutations significantly enriched in negative neo-peptides included serine to arginine (S → R, OR = 0.20, p-adj = 0.008) and arginine to cysteine (R → C, OR = 0.59, p-adj = 0.003).

To refine our analysis, we examined the physicochemical properties affected by mutations (Fig. 8B). Acidic → basic mutations showed the most significant enrichment in neo-epitopes (OR = 1.62, p-adj = 6.49 × 10⁻^5^). Other enriched transitions included non-aromatic → aromatic (OR = 1.22, p-adj = 0.005) and non-aliphatic → aliphatic mutations (OR = 1.19, p-adj = 0.011), both of which increase hydrophobicity. The enrichment of aromatic substitutions aligns with prior findings that aromatic side chains enhance the stability of MHC–peptide complexes [23].

Conversely, mutations that did not alter physicochemical properties (i.e., "No change"; OR = 0.81, p-adj = 0.001) were enriched in negative neo-peptides, highlighting the importance of biochemical changes in neo-epitope recognition. The transitions from non-small to small residues (OR = 0.83, p-adj = 0.038) and from basic to neutral residues (OR = 0.84, p-adj = 0.038) were also enriched in negative neo-peptides, suggesting that reduced side-chain bulkiness or lost electrostatic interactions may hinder epitope recognition.

## Discussion

This study presents the largest systematic meta-analysis of experimentally tested neo-peptides to date, integrating over 16,000 neo-peptides tested in more than 20,000 T cell assays across 180 studies. We identified key features that distinguish neo-epitopes from non-immunogenic neo-peptides and uncovered biases in the literature.

ELISPOT assays measuring IFN-γ production were predominantly used for evaluating neo-epitope T cell recognition. The limited assessment of other cytokines (e.g., IL-2 and TNF-α) may overlook polyfunctional T cell responses critical for durable immunity [24], skewing our understanding of neo-epitope immunogenicity, and potentially underestimating the therapeutic potential of neo-epitopes that elicit broader cytokine profiles. Future studies should diversify the assay types to capture a more comprehensive immunological landscape.

Our finding that driver genes are enriched for neo-epitopes, despite weaker correlations with protein length, challenges the idea that neo-epitope generation is solely a stochastic process tied to mutation burden. Previous work suggested that mutations in driver genes produce more recognizable peptides due to their oncogenicity and that evolutionary pressure impacts both their tumor-promoting capacity and their ability to evade immune surveillance [25]. Previous studies also suggested that clonality is important for immune recognition and showed that clonal neo-epitopes correlate with immunotherapy response [26]. Furthermore, previous studies showed that neo-epitope immunogenicity is shaped by the expression levels of their source proteins and genes [27–31]. Driver mutations are clonal and present in many tumor cells, often highly expressed, enhancing immune recognition [32]. These findings have important implications for neo-epitope prioritization, as targeting driver-derived neo-epitopes could simultaneously address immunogenicity and tumor fitness [33, 34].

However, these results may be biased due to the more frequent testing of neo-peptides derived from driver gene mutations, increasing the likelihood of validating a neo-epitope. This bias was particularly evident in with TP53 where we found a strong correlation between mutation frequency and study coverage. This finding highlights a research bias toward frequent mutations, leaving a subset of recurrent but understudied mutations as untapped opportunities for novel neo-epitope discovery.

The variation in neo-epitope fractions across cancer types aligns with their known immunotherapy responsiveness [14]. Skin and lung cancers exhibited the highest neo-epitope fractions and respond well to immunotherapy, while colorectal and esophageal cancers had the lowest fractions and are less responsive to immunotherapy. Normalization by mutation burden revealed that the low neo-epitope positivity in colorectal cancer may stem from dilution by a high number of non-immunogenic mutations rather than a lack of immunogenic potential. Interestingly, bile duct and pancreatic cancers, although ‘cold’ tumors with low mutation rates, showed a higher proportion of neo-epitopes, suggesting that even their few mutations can generate functional neo-epitopes. This observation is supported by recent findings demonstrating that functionally immunogenic mutations can indeed be identified in cancers with low mutational burden at a frequency higher than previously appreciated [35]. However, interpretation of cancer-type-specific positivity rates should be approached with caution. Cancer types with limited neo-peptide testing may exhibit inflated positivity rates if only high-confidence or previously validated peptides were assayed. For example, tumor types such as urinary bladder and appendix cancer exhibited 100% positivity rates, which are likely influenced by small sample sizes and targeted study designs rather than true biological enrichment.

An important limitation of this analysis was the lack of microsatellite instability (MSI) status in CEDAR. MSI-high (MSI-H) colorectal cancers are more immunogenic than microsatellite stable (MSS) tumors, which generally show lower responsiveness to immunotherapy [36]. The reported low neo-epitope positivity of 6% likely averages both subtypes, potentially underestimating MSI-H cases. A similar issue exists for lung cancer; while there is some distinction between non-small cell lung cancer (NSCLC) subtypes, detailed subtype annotations were not available for all neo-peptides. Additionally, the absence of patients’ smoking history is significant, as it can influence mutation burden and immunogenicity. Smoking-associated cancers generally have higher mutational loads and more neo-epitope due DNA damage from tobacco exposure, promoting T cell recognition [37, 38]. In contrast, never-smoker cancers are usually characterized by driver mutations, exhibit lower mutation burden, and show reduced responsiveness to immunotherapy [39, 40]. Without stratification by subtype or smoking status, the observed lung cancer results likely reflect a blended signal across immunologically distinct subgroups.

Our analysis highlighted that neo-epitopes have primarily been studied in the context of HLA class I, underlining the important role of CD8⁺ T cell responses. However, a higher fraction of neo-epitopes was presented by HLA class II, suggesting that CD4⁺ T cells are underexplored. Both preclinical and clinical studies highlighted the critical role of HLA class II-restricted neo-epitopes, demonstrating that CD4⁺ T cell help enhances response to checkpoint blockade therapy and neo-epitope vaccination [41, 42]. Another study showed that a small number of CD4⁺ T cells can eradicate MHC-deficient tumors that escape CD8⁺ T cell targeting [43]. A key limitation of the HLA restriction data analyzed here is that only 31% of the assays included HLA allele information with four-digit resolution, limiting a comprehensive assessment of HLA-binding preferences. It is important to interpret HLA enrichment results with caution due to potential sampling biases. Common alleles, such as HLA-A*02:01, are more frequently studied, which may lead to a saturation effect where most immunogenic peptides have already been identified and additional testing yields predominantly negative results. Conversely, rare HLA alleles may be tested only when immunogenicity is suspected or already demonstrated, leading to inflated positivity rates. This bias may also contribute to the observed enrichment of neo-epitopes among HLA class II alleles, which are generally less frequently tested but often in preselected or context-specific settings.

We found that amino acid substitutions, like S → F and E → K, are enriched in neo-epitopes, indicating these mutations enhance T cell recognition by improving hydrophobicity and altering charge. This is consistent with reports highlighting the role of hydrophobicity in stabilizing peptide-MHC complexes [44, 45] and findings on charge-dependent immunogenicity [23, 46]. Conversely, substitutions like S → R and R → C were enriched in negative neo-peptides, indicating reduced T cell recognition, consistent with findings that altered charge can negatively impact peptide stability or presentation efficiency [23]. However, the implications of cysteine residues require caution, as they can cause oxidation and disulfide bond formation, affecting MHC binding and peptide synthesis [47, 48]. Furthermore, our analysis did not consider structural or conformational changes, which are also important for T cell recognition and immune response [49].

Neo-epitopes can arise from various genomic and transcriptomic alterations, including frameshift mutations, gene fusions, and RNA alteration events. However, this meta-analysis focused exclusively on neo-peptides derived from SNVs. Other mutation types involve more complex curation procedures and might be incomplete at this point. We are actively working to improve the accuracy of curated neo-epitopes in CEDAR derived from all mutation types. Notably, frameshift-derived neo-peptides may lead to the generation of identical peptide sequences from distinct mutations across patients, making them especially attractive for shared antigen targeting [50, 51].

A key limitation of our analysis concerns the reliability of negative results in neo-epitope annotation. Unlike infectious disease settings, where immunogenicity is defined through repeated testing across multiple donors and time points, neo-peptides are typically tested in a single patient at a single time. This limited framework increases the risk of false negatives, as neo-peptides may be immunogenic but remain unvalidated due to assay constraints. Previous work demonstrated that many neo-epitopes labeled as negative were later shown to elicit T cell responses when retested under different conditions [52]. In addition to technical constraints, T cell-intrinsic factors also contribute to false-negative outcomes. Tumor-reactive T cells isolated from cancer patients often exhibit features of exhaustion or dysfunction due to chronic antigen exposure in the tumor microenvironment [53]. These exhausted T cells may have reduced cytokine production or proliferative capacity, limiting their detectability in standard functional assays. As a result, truly immunogenic neo-epitopes may be misclassified as non-immunogenic due to attenuated T cell responses.

Similarly, we defined a neo-peptide as a neo-epitope based on a single positive assay; however, immunogenicity is context-dependent and varies across cancer types and patients, likely due to differences in expression levels, MHC presentation machinery, immune infiltration, or T cell diversity. This variability complicates the definition of a “true” neo-epitope. Our meta-analysis also combined data generated from assays with variable setups and readouts, introducing heterogeneity that can obscure subtle biological signals. These limitations do not undermine the value of this meta-analysis but rather highlight the complexity of neo-epitope biology and the challenges in defining and comparing heterogeneous datasets.

Together, these findings underscore the value of CEDAR as a centralized and richly annotated resource for exploring neo-epitopes. By systematically aggregating and analyzing experimentally tested neo-peptides, this meta-analysis revealed both prevailing patterns and critical gaps in the field. As CEDAR continues to expand in scope and depth, it can serve as a powerful platform for guiding neo-epitope prioritization and accelerating the development of personalized cancer immunotherapy.

## Methods

### Retrieving the dataset of experimentally tested neo-peptides from CEDAR

In January 2025, we downloaded T cell assay data from the CEDAR Exports page (https://cedar.iedb.org/database_export_v3.php). We filtered for entries annotated as “neo-epitope” in the “Epitope Relation” field and further refined the dataset to include only neo-peptides derived from single nucleotide variations (SNVs) by comparing the neo-peptide sequence in the “Epitope_Name” field to the wild-type sequence in the “Related Object_Name” field. To restrict the data to humans, we filtered for entries labeled "Homo sapiens (human)" in the “Host_Name” and “Related Object_Source Organism” fields. We included only assays associated with cancer diagnoses by examining the “1st in vivo Process_Disease” and “2nd in vivo Process_Disease” fields and removing any entries with “healthy.” Additionally, we eliminated entries that used cell lines as antigen-presenting cells by filtering out those labeled as “Cell Line” in the “Antigen Presenting Cell_Culture Condition” field.

Curation is done on a per-publication basis, leading to multiple studies reporting the same peptide with varying results. A neo-peptide was classified as a neo-epitope (considered “positive”) if at least one T cell assay showed a positive response. Neo-peptides with no positive assay results were categorized as “negative.” This was based on the “Assay_Qualitative Measurement” field, where outcomes of “low positive,” “medium positive,” or “high positive” were deemed positive.

### Cancer type mapping

Neo-peptides were classified into cancer types using primary disease annotations from CEDAR. To improve standardization, a hierarchical approach linked specific cancer subtypes (e.g., colorectal adenocarcinoma) to broader categories (e.g., colorectal cancer). This was done by navigating the Disease Ontology [54] and mapping broader entries to their subtypes with custom Python scripts.

### Compiling a dataset of somatic mutations from cBioPortal

We obtained a dataset of somatic mutations in cancer samples from cBioPortal to analyze mutational frequencies across different cancer types and antigens. Using the curated set of 226 non-redundant studies (including TCGA and non-TCGA) without overlapping samples, we excluded studies with targeted sequencing, pediatric tumors, and cancer cell lines. We focused on studies providing clinical data for profiled patients, particularly the cancer type.

To assess mutational frequency, we calculated the percentage of samples with mutations in each gene. To evaluate mutational burden by cancer type, we determined the median number of mutations per type. The fraction of positive neo-epitopes per cancer type was normalized using a specific formula:

#### $${normalized fraction of neo-epitopes}_{cancer type}=\frac{total number of positive neo-epitopes in cancer type}{median number of mutations in cancer type from cBioPortal*100}$$.

The resulting value was then scaled to fall into a range between 0 and 1 using the following formula: $$\frac{{normalized fraction of neo-epitopes}_{cancer type}-\text{min}(normalized fraction of neo-epitopes)}{\text{max}\left(normalized fraction of neo-epitopes\right)-\text{min}(normalized fraction of neo-epitopes)}$$. The resulting values were converted into percentages.

### Classification of amino acid physicochemical properties

The physicochemical properties of the amino acids were classified based on the established biochemical literature. Polarity and charge were assigned using hydropathy scales and ionization studies [55, 56]. Acidity classifications were determined based on the pKa values [57]. Aromaticity and aliphaticity were categorized according to the side-chain composition [58]. Amino acid size distinctions were derived from systematic evaluations of residue volume and steric effects [56]. These classifications were used to assess physicochemical changes introduced by mutations and their association with neo-epitopes.

### Statistical methods

All statistical analyses were performed in R (version 4.3.1) using various packages for data manipulation, visualization, and statistical testing. Correlation analyses were conducted using Pearson’s correlation coefficient (R) computed using the cor.test() function. Enrichment analyses were performed using Fisher’s exact test with the fisher.test() function, with multiple hypothesis testing corrections applied using the Benjamini-Hochberg (BH) method with the function p.adjust(). To ensure reproducibility, all R scripts were executed in a controlled environment, and session information was recorded using sessionInfo().

## Supplementary Information

Below is the link to the electronic supplementary material.

Supplementary file1 (DOCX 1654 KB)

Supplementary file2 (XLSX 10973 KB)

Supplementary file3 (XLSX 1307 KB)

Supplementary file4 (XLSX 11 KB)

Supplementary file5 (XLSX 8 KB)

Supplementary file6 (XLSX 420 KB)

Supplementary file7 (XLSX 21 KB)

Supplementary file8 (XLSX 14 KB)

Supplementary file9 (XLSX 24 KB)

Supplementary file10 (XLSX 14 KB)

Supplementary file11 (XLSX 8 KB)

Supplementary file12 (XLSX 10 KB)

Supplementary file13 (XLSX 987 KB)

## Data Availability

Research data supporting this publication are available from the Cancer Epitope Database and Analysis Resource (CEDAR) located at https:/cedar.iedb.org.

## Abbreviations

AA: Amino acid
BH: Benjamini-Hochberg
CEDAR: Cancer Epitope Database and Analysis Resource
cBioPortal: CBio Cancer Genomics Portal
DAI: Differential Agretopicity Index
ELISA: Enzyme-Linked Immunosorbent Assay
ELISPOT: Enzyme-Linked ImmunoSpot
EL %Rank: Eluted Ligand Percentile Rank
HLA: Human Leukocyte Antigen
ICS: Intracellular Cytokine Staining
IEDB: Immune Epitope Database
IFN-γ: Interferon Gamma
IL-2: Interleukin-2
IL-5: Interleukin-5
IL-17: Interleukin-17
MHC: Major Histocompatibility Complex
MHCLE: MHC Ligand Elution
MSI: Microsatellite Instability
MSI-H: Microsatellite Instability–High
MSS: Microsatellite Stable
NSCLC: Non-Small Cell Lung Cancer
OR: Odds ratio
p-adj: Adjusted p-value
SNV: Single nucleotide variant
TNF-α: Tumor Necrosis Factor Alpha
