# Supplementary material for: A meta-analysis of experimentally validated neo-epitopes: patterns, biases, and opportunities
Source: Cancer Immunol Immunother. 2025 Nov 6;74(12):362. doi: 10.1007/s00262-025-04209-7 (PMC12592574; doi:10.1007/s00262-025-04209-7)
Supplement: Supplementary file 5 — Supplementary file5 (XLSX 8 KB) [file 262_2025_4209_MOESM5_ESM.docx]

**Supplementary Tables**

Table S1: All neo-peptide and neo-epitope data downloaded from CEDAR on assay level

Table S2: All neo-peptide and neo-epitope data on peptide level. Peptides meeting stricter reproducibility criteria, defined as having results from at least three reported T cell assays and classified as neo-epitopes only if at least two assays showed positive responses, were flagged in the dataset.

Table S3: A random subset of neo-peptides was reviewed to assess variability in reported experimental data.

Table S4: Overview of assay types

Table S5: Overview of source proteins

Table S6: TP53-derived neo-peptides and neo-epitopes

Table S7: Ras-derived neo-peptides and neo-epitopes

Table S8: Overview of repeatedly tested mutations

Table S9: Investigator Overlap Across Studies Testing the Same Mutations.

Table S10: Overview of neo-peptides and neo-epitopes across cancer types

Table S11: Shared neo-peptides and neo-epitopes across cancer types

Table S12: NetMHCpan predictions


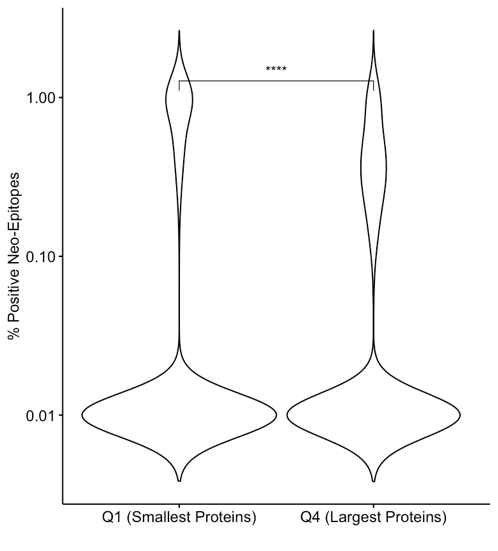


Figure S1: Fraction of neo-epitopes in smallest vs largest proteins. Fraction of neo-epitopes per tested neo-peptide was calculated for each protein and compared across protein length quartiles. Proteins in the smallest quartile (Q1) showed a higher mean neo-epitope fraction (0.1334) than those in the largest quartile (Q4, 0.1176; Wilcoxon rank sum test, p = 5.55 × 10⁻⁴).


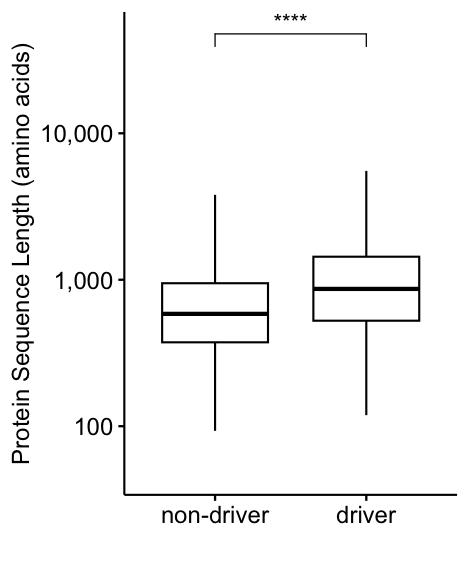


Figure S2: Sequence length of proteins encoded by driver vs. non-driver genes. Protein lengths of genes classified as cancer drivers and non-drivers were compared. Proteins encoded by driver genes had a significantly greater median length (866 amino acids) than those encoded by non-driver genes (585 amino acids; Wilcoxon rank sum test, p < 2.2 × 10⁻¹⁶).


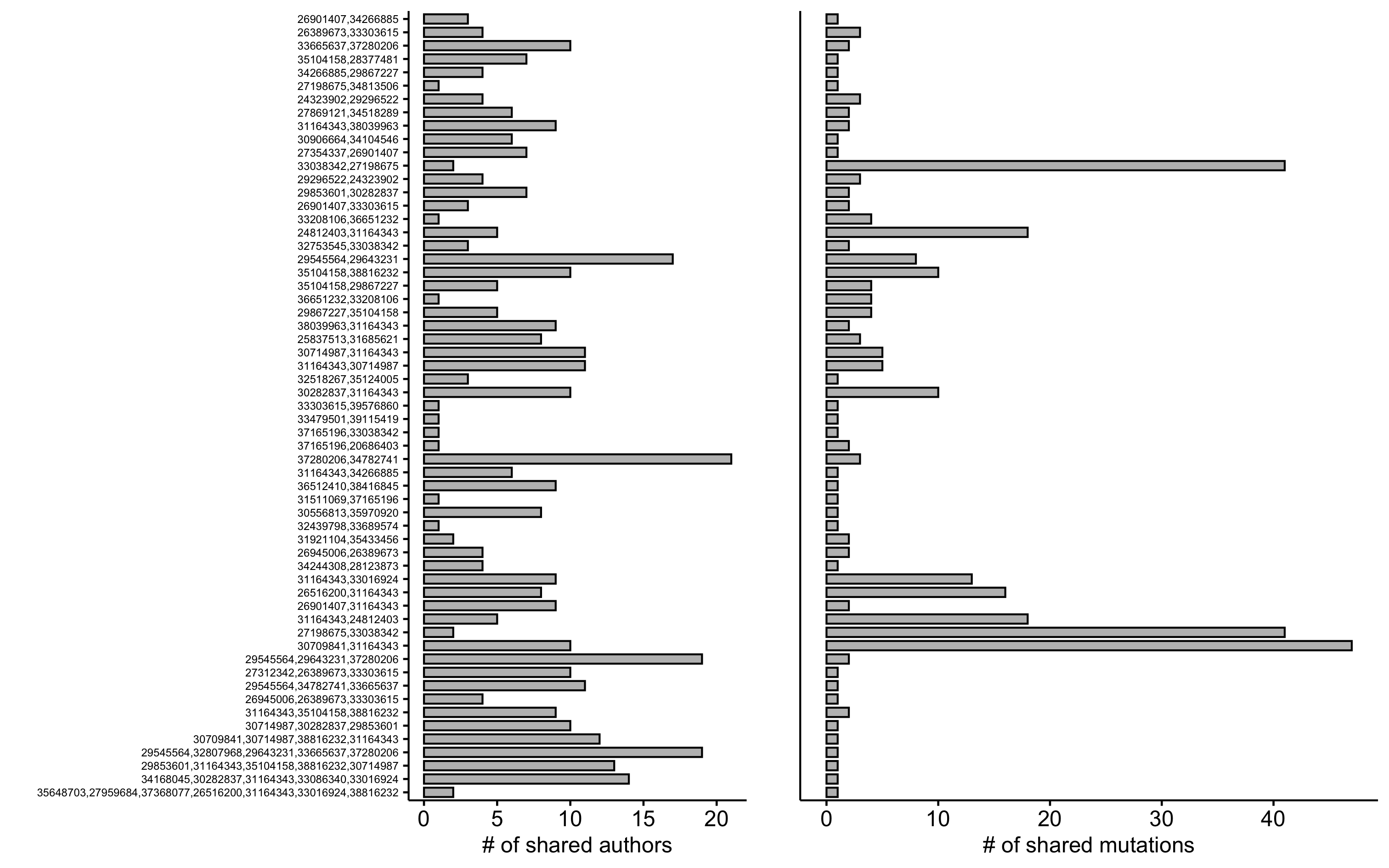


Figure S3: Author overlap was analyzed across 63 unique study groups, defined by distinct combinations of PubMed IDs that tested the same mutations. For each group, the number of shared authors and the number of distinct mutations tested were quantified.


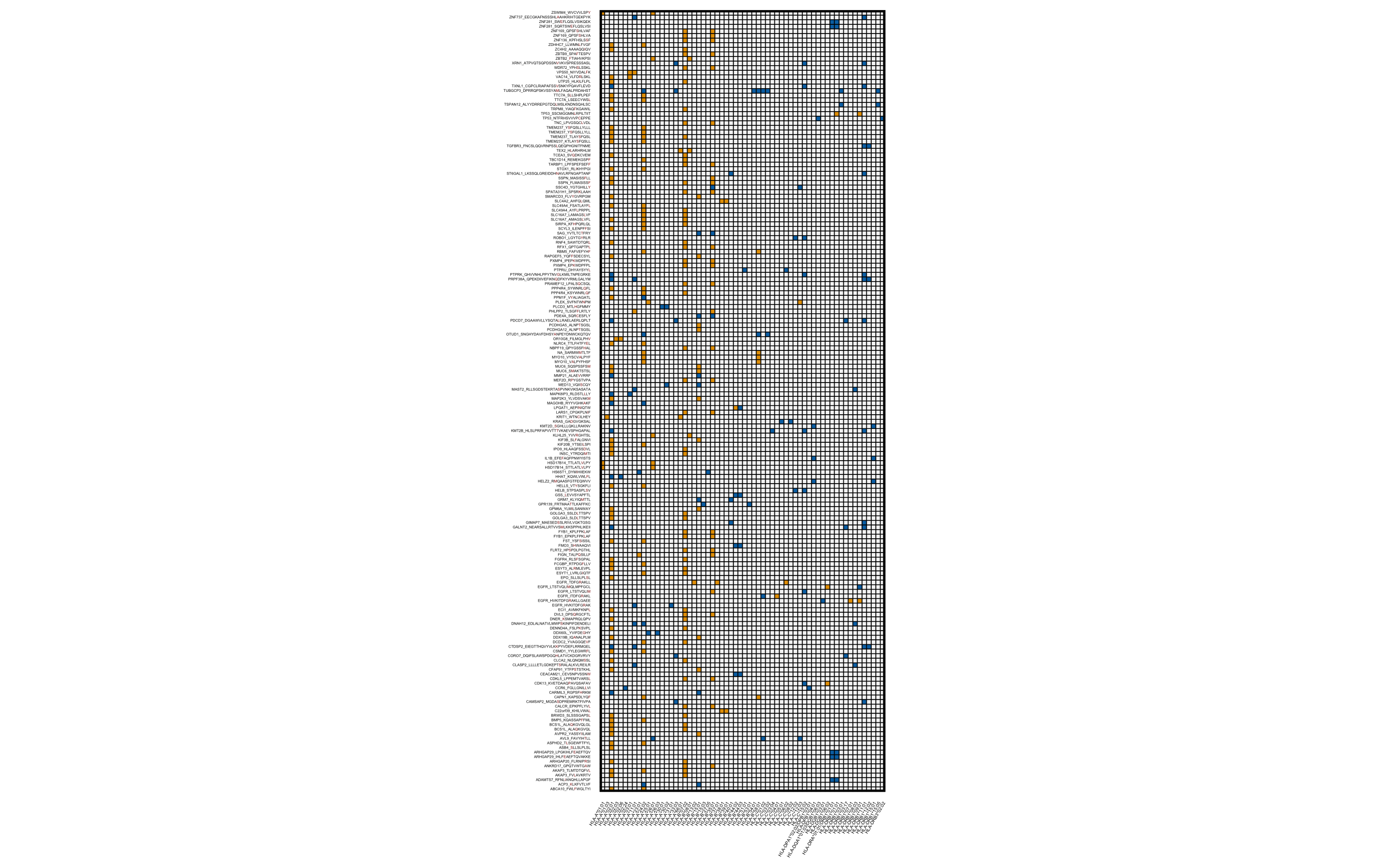


Figure S4: Overview of all shared neo-peptides between HLA alleles. Neo-peptides tested in the context of at least two distinct HLA alleles were identified (n = 166).


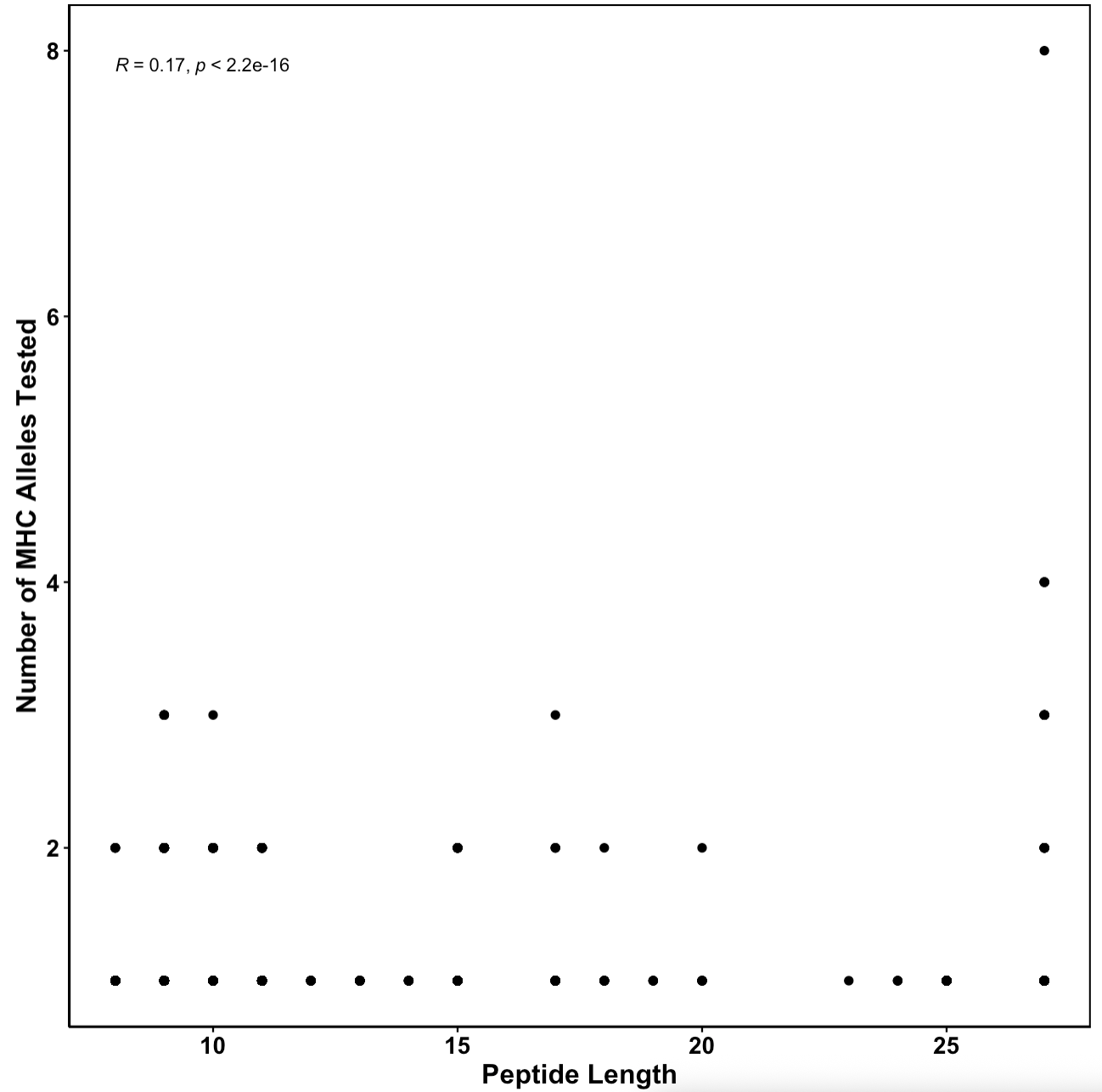


Figure S5: Correlation between peptide length and number of MHC alleles tested. Peptide length was compared to the number of distinct HLA alleles tested per peptide. A positive correlation was observed (Pearson correlation coefficient = 0.17, p = 2.2 × 10⁻¹⁶).


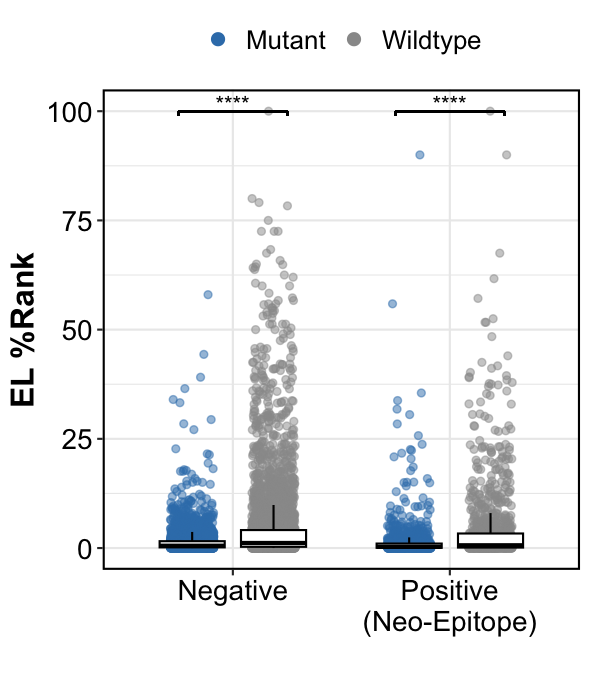


Figure S6: NetMHCpan EL %-Rank predictions for mutant and wild-type peptides of negatives and positives. Comparison of EL %-Rank binding predictions between mutant and wild-type peptide pairs. Mutant peptides showed significantly stronger predicted binding than their wild-type counterparts in both neo-epitope and negative neo-peptide groups (Wilcoxon test, p < 2.2 × 10⁻¹⁶).


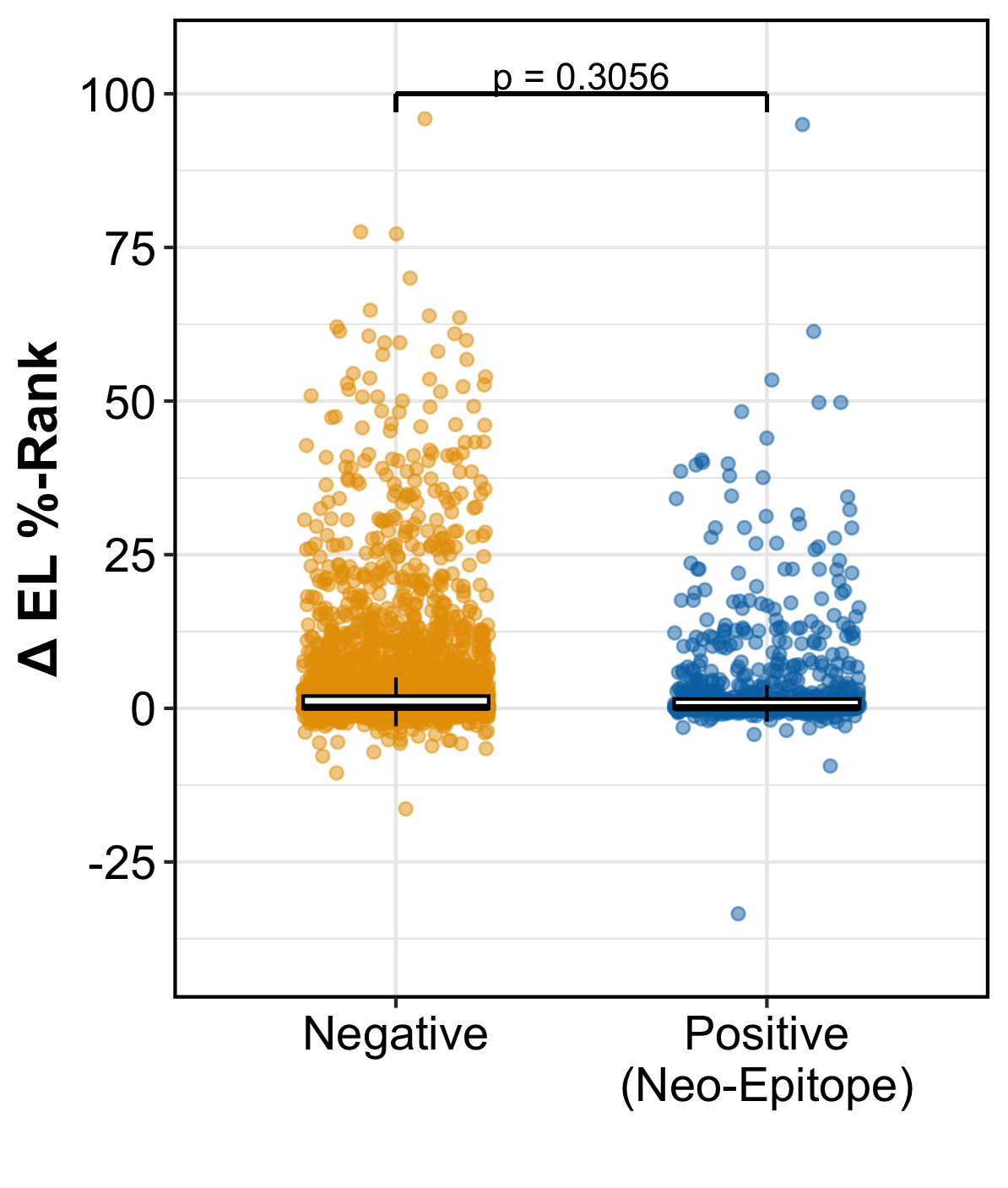


Figure S7: Differential Agretopicity Index (DAI), defined as the difference in binding affinity between mutant and wild-type peptides, for positives and negatives. DAI scores were calculated as the difference in EL %-Rank between mutant and wild-type peptides. No significant difference in DAI scores was observed between neo-epitopes and negative peptides (Wilcoxon test, p = 0.3056).
